# First case of *TREX1* mutation-driven retinal vasculopathy with cerebral leukoencephalopathy and systemic manifestations coexisting with lupus nephritis: a case report and mechanistic discussion

**DOI:** 10.3389/fimmu.2026.1758743

**Published:** 2026-03-19

**Authors:** Wenjie Hao, Shu Zhai, Qianqian Zhu, Xuan Zhou, Tingting Shen, Wei He, Yuying Sun, Wenming Yang, Yulong Yang

**Affiliations:** 1 Department of Neurology, The First Affiliated Hospital of Anhui University of Chinese Medicine, Hefei, Anhui, China; 2 Key Laboratory of Xin’An Medicine, Ministry of Education, Hefei, Anhui, China; 3 Center for Xin’an Medicine and Modernization of Traditional Chinese Medicine, Institute of Health and Medicine Hefei Comprehensive National Science Center, Hefei, Anhui, China; 4 Wangjing Hospital, China Academy of Chinese Medical Sciences, Beijing, China; 5 Intensive Care Unit, The First Affiliated Hospital of Anhui University of Chinese Medicine, Hefei, Anhui, China; 6 Department of Pathology, The First Affiliated Hospital of Anhui Medical University, Hefei, Anhui, China

**Keywords:** case report, lupus nephritis, retinal vasculopathy with cerebral leukoencephalopathy and systemic manifestations (RVCL-S), systemic lupus erythematosus, TREX1 gene

## Abstract

**Background:**

The *TREX1* gene is fundamental for the removal of cytosolic DNA and the preservation of immune tolerance. Mutations within this gene are implicated in a range of disorders, such as Retinal Vasculopathy with Cerebral Leukoencephalopathy and Systemic Manifestations (RVCL-S), Aicardi-Goutières syndrome (AGS), familial chilblain lupus, and systemic lupus erythematosus (SLE). To the best of our knowledge, this report documents the rare coexistence of *TREX1*-associated RVCL-S and biopsy-proven lupus nephritis in a single patient.

**Case presentation:**

We report a case involving a middle-aged woman diagnosed with lupus nephritis, confirmed through renal biopsy, who later experienced progressive neurological deficits. Brain imaging demonstrated typical signs of cerebral leukoencephalopathy. Whole-exome sequencing uncovered a pathogenic *TREX1* mutation (c.811_812dup, p.Asp272Argfs*6), resulting in a diagnosis of genetically confirmed *TREX1*-associated RVCL-S with an incomplete phenotype, coexisting with lupus nephritis. This case underscores a significant clinical challenge: the use of immunosuppressive therapy for lupus nephritis is hypothesized to potentially exacerbate the vascular complications associated with RVCL-S.

**Conclusion:**

To the best of our knowledge, this case represents the first documented instance of the *TREX1* p.Asp272Argfs*6 mutation contributing to the coexistence of RVCL-S and lupus nephritis. This discovery broadens the recognized phenotypic spectrum associated with *TREX1*-related disorders and underscores a distinct therapeutic challenge in their management. It is important for clinicians to be aware of this new phenotype to ensure prompt diagnosis and tailored treatment strategies.

## Introduction

Retinal vasculopathy with cerebral leukoencephalopathy and systemic manifestations (RVCL-S) is an extremely rare autosomal dominant small-vessel vasculopathy caused by mutations in the three-prime repair exonuclease 1 (*TREX1*) gene (1, 2). To date, about 50 families and 200 patients have been reported worldwide; disease onset usually occurs after 40 years of age, with a mean survival of only around 9 years after diagnosis. RVCL-S mainly affects highly vascularized organs such as the retina, brain, kidneys and liver, leading to multi-organ dysfunction and premature death. Its pathophysiology is driven by *TREX1* mutations that induce vascular endothelial dysfunction, likely through endoplasmic reticulum stress and altered glycosylation, and clinically manifests as retinal vasculopathy, cerebral leukoencephalopathy, hepatic dysfunction, renal impairment and hematological abnormalities. The understanding and nomenclature of this condition have evolved over time: it was first described in 1988 as “cerebroretinal vasculopathy,” later termed “hereditary vascular retinopathy” in 1991 and “hereditary endotheliopathy with retinopathy, nephropathy and stroke” in 1997. A major advance came in 2007, when Richards et al. identified heterozygous *TREX1* mutations as the common genetic basis for these entities (2). In 2016, Anine H. Stam and colleagues analyzed 11 families with 78 mutation carriers and proposed the unified designation “RVCL-S,” which is now widely adopted (3).

Systemic lupus erythematosus (SLE) is a heterogeneous systemic autoimmune disorder characterized by immune dysregulation arising from the interplay of genetic predisposition, environmental exposures and stochastic events (4, 5). In 2012, Belot formally introduced the concept of “monogenic lupus,” referring to SLE subtypes driven by single-gene mutations (6). Mutations in *TREX1* are among the most frequent causes of monogenic lupus in adults, being identified in approximately 0.5–2% of screened cases (7–9). Lupus nephritis (LN) is a common and severe manifestation of SLE caused by the deposition of immune complexes in the kidneys and represents a major contributor to SLE-related mortality (10).

The *TREX1* gene encodes a major 3′–5′ DNA exonuclease that degrades cytosolic DNA to maintain immune tolerance. While N-terminal mutations typically cause systemic lupus erythematosus (SLE) or Aicardi-Goutières syndrome via loss of enzymatic function, C-terminal frameshift mutations are uniquely associated with RVCL-S through protein mislocalization (1, 11). To the best of our knowledge, a comprehensive literature review across major medical databases (e.g., Web of Science, PubMed) revealed no prior documentation of *TREX1*-associated RVCL-S co-occurring with biopsy-proven lupus nephritis (LN). Notably, only a single case of RVCL-S overlapping with SLE has been previously described (12). Here, we report the first such case, associated with a *TREX1* p.Asp272Argfs*6 mutation, to raise awareness of this distinct overlapping phenotype.

## Case presentation

A middle-aged woman was admitted to the Department of Neurology in August 2024 with a 3-year history of insidiously progressive right lower limb weakness and gait instability. She also reported blurred vision and memory decline. Prior to admission, an outpatient brain MRI revealed confluent periventricular and deep white matter lesions with demyelination, multiple lacunar infarcts, intracranial calcifications, and cerebral atrophy. She was admitted for further evaluation of leukoencephalopathy of unknown etiology.

**Figure 1:**
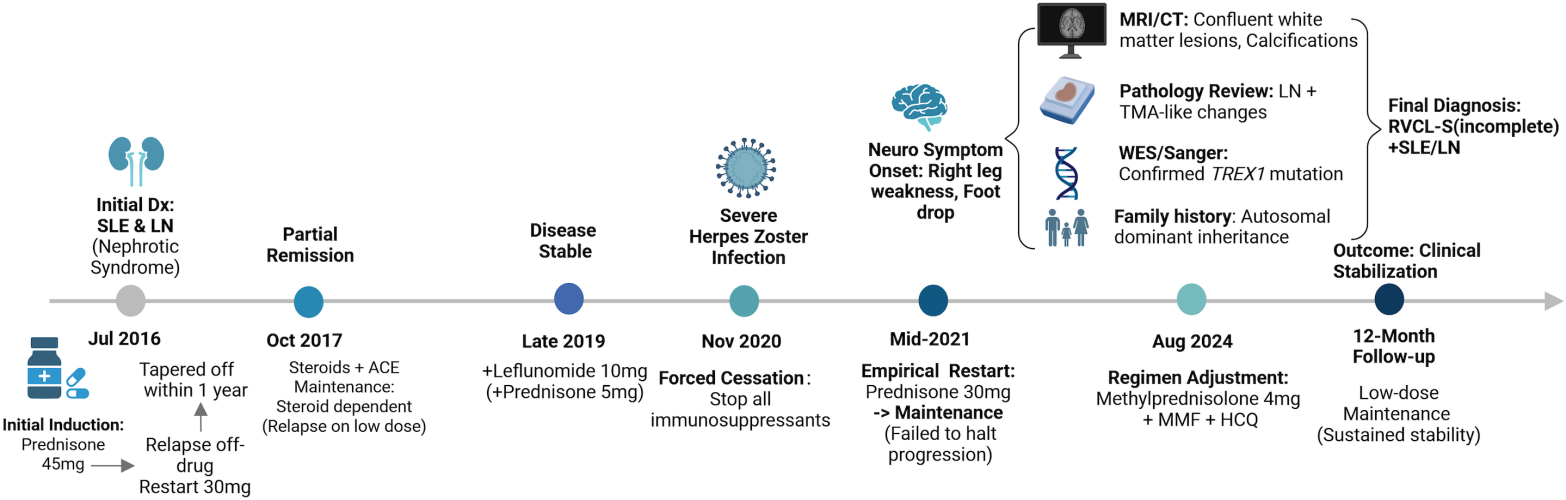
Clinical timeline and therapeutic management trajectory. The timeline illustrates the disease course from the initial diagnosis of SLE and Lupus Nephritis (2016) to the current neurological status (2024). The upper section marks key clinical events, including the severe herpes zoster infection (Nov 2020) that necessitated the cessation of immunosuppression. The lower section details the medication adjustments, highlighting the correlation between treatment withdrawal and the onset of neurological symptoms. Dx, diagnosis; LN, lupus nephritis; WES, whole-exome sequencing; RVCL-S, retinal vasculopathy with cerebral leukoencephalopathy and systemic manifestations; MMF, mycophenolate mofetil; HCQ, hydroxychloroquine. Created with BioRender.com.

Past Medical History and Disease Evolution: Her medical history is complex, spanning nine years (**Figure 1**).

Renal-Dominant Phase (2016-2020): She was first diagnosed with Systemic Lupus Erythematosus (SLE) and Lupus Nephritis (LN), manifesting as nephrotic syndrome, in July 2016 at an external hospital. Initial induction therapy with oral prednisone (45 mg/d) was tapered and discontinued within one year as proteinuria resolved. However, in mid-2017, proteinuria recurred two months after cessation, prompting a re-escalation of prednisone to 30 mg/d. By October 2017, partial remission was achieved with corticosteroids combined with an ACE inhibitor. During the maintenance phase, she exhibited clear steroid dependence, flaring whenever prednisone was tapered to 5–10 mg/d. Leflunomide (10 mg/d) was added in late 2019 in combination with prednisone (5 mg/d), resulting in clinical stability. In November 2020, she developed a severe herpes zoster infection; consequently, immunosuppressants were tapered and discontinued to manage the infection. Notably, during this initial phase, neurological examination was unremarkable, with no signs of motor weakness, gait instability, or pathological reflexes.

Neuro-Symptomatic Phase (2021-Present): Approximately six months after the cessation of immunosuppression (mid-2021), she insidiously developed right lower limb weakness, foot drop, and a dragging gait without identifiable trauma. External electromyography (EMG) indicated right peroneal and tibial nerve damage. Given the suspicion of peripheral neuropathy or active lupus, oral prednisone was empirically restarted at 30 mg/d in mid-2021, and subsequently adjusted to a maintenance regimen (prednisone 5 mg/d, mycophenolate mofetil 0.5 g/d, and hydroxychloroquine 200 mg/d). Despite re-initiating immunosuppression, her neurological deficits progressively worsened until the current admission.

Physical Examination At admission, the patient exhibited moderate cognitive impairment (MoCA score 13/30). Cranial nerve examination was unremarkable. Motor examination revealed significant abnormalities: spasticity in the right lower limb with grade 4/5 power, hyperreflexia (4+), and sustained ankle clonus. Cerebellar testing showed impaired rapid alternating movements and dysmetria on the right. Bilateral pes cavus was noted (**Supplementary Figure 1**). Ophthalmological evaluation, including dilated fundus examination, was performed at admission and revealed no signs of retinal vasculopathy, hemorrhage, or cotton-wool spots (**Supplementary Figure 2**). Although the patient complained of blurred vision, given the normal funduscopic findings, this deficit is likely central (cortical) or refractive in nature. Unfortunately, the patient declined repeat dilated eye examinations during follow-up.

### Diagnostic workup

**Figure 2:**
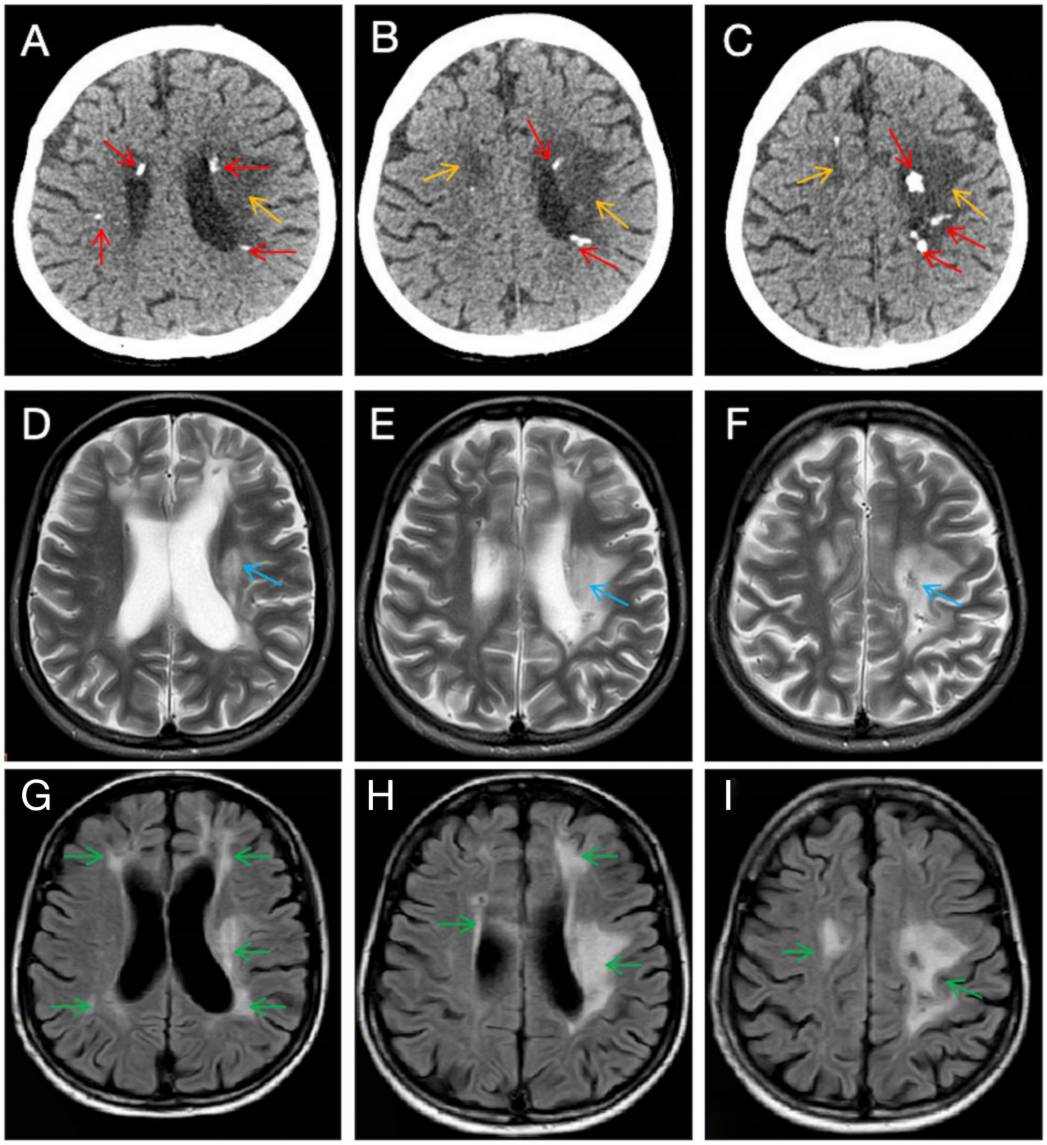
Neuroimaging features of the patient. **(A–C)** Non-contrast head CT scan reveals extensive hypodense lesions in the periventricular regions [orange arrows, **(A, B)**] and centrum semiovale [orange arrow, **(C)**], accompanied by multiple calcifications [red arrows, **(A–C)**]. **(D–F)** T2-weighted MRI shows non-specific T2 hyperintense white matter lesions (blue arrows) and cerebral atrophy. **(G–I)** FLAIR MRI demonstrates extensive, asymmetrical, confluent lesions involving the periventricular regions, frontal, and parietal lobes (green arrows).

Neuroimaging: Repeat MRI and CT confirmed confluent periventricular white matter demyelination, multiple lacunar infarcts, and characteristic intracranial calcifications (**Figure 2**). This pattern is highly suggestive of a hereditary vasculopathy rather than typical inflammatory demyelination.

**Figure 3:**
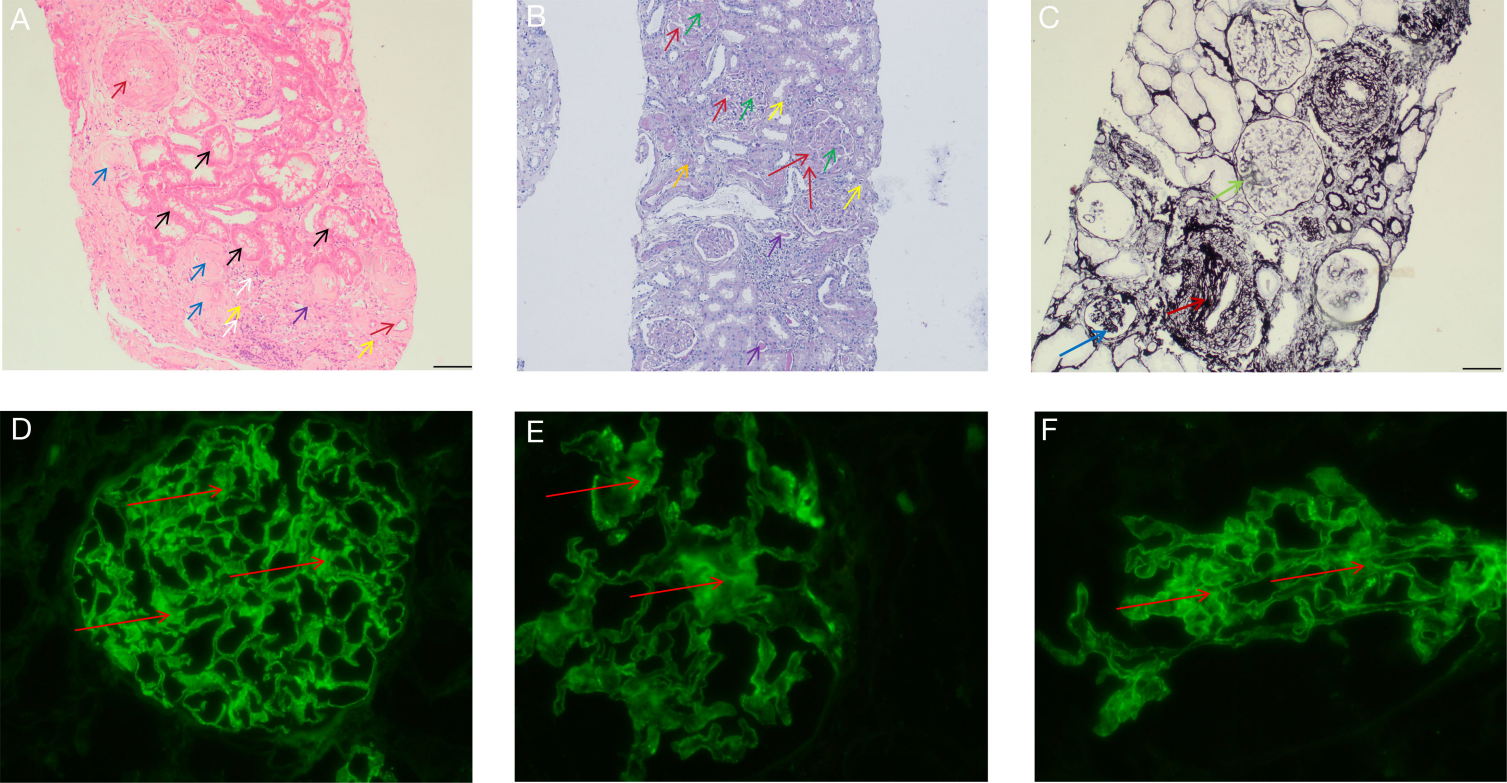
Renal pathological findings. **(A)** Hematoxylin and eosin (H&E) stain (×100 magnification) shows multiple sclerotic glomeruli (blue arrow), intimal thickening of the vascular wall (crimson arrow), focal tubular coagulative necrosis (black arrow), inflammatory cell infiltration (white arrow), vacuolar degeneration of tubular epithelial cells (yellow arrow), and casts within renal tubules (purple arrow). **(B)** Periodic acid-Schiff (PAS) stain (×100 magnification) demonstrates thickened glomerular capillary basement membranes (crimson arrow), mesangial matrix expansion (green arrow), vacuolar degeneration of tubular epithelial cells (yellow arrow), casts within tubules (purple arrow), and intimal thickening of a small renal artery (orange arrow). **(C)** Periodic acid-Schiff methenamine silver (PASM) stain (×100 magnification) reveals a double-contour appearance of the glomerular capillary basement membrane (light green arrow), segmental luminal narrowing due to endocapillary hyperplasia (dark blue arrow), and intimal thickening of the vascular wall (crimson arrow). **(D–F)** Immunofluorescence microscopy shows diffuse global granular deposits along capillary loops and in the mesangium (red arrows): **(D)** IgG (intensity: +++). **(E)** Kappa light chain (intensity: ++). **(F)** Lambda light chain (intensity: ++). Additionally, the patient exhibited granular deposits of IgA (+), IgM (±), C3 (+), and C1q (±); representative images are provided in **Supplementary Figure 3**.

Laboratory and Pathology Review: Autoimmune antibody screening on this admission was negative (detailed laboratory data are provided in **Supplementary Table 1**). A review of records from August 2021 (external hospital) revealed renal insufficiency (Creatinine 118.1 µmol/L) and immunological activity (ANA positive 1:320, low C3/C4) at that time. Re-evaluation of her previous renal biopsy (**Figure 3**) confirmed ISN/RPS Class IV Lupus Nephritis (LN). According to the NIH scoring system, the Activity Index (AI) was 8/24, characterized by segmental endocapillary hypercellularity and “wire-loop” lesions, while the Chronicity Index (CI) was 5/12, indicating established glomerular sclerosis and interstitial fibrosis. Notably, the biopsy revealed specific features suggestive of a thrombotic microangiopathy (TMA)-like process, including double-contouring of the glomerular capillary basement membrane and ischemic glomerular shrinkage. These findings coexisted with diffuse granular deposits of IgG (+++), C3 (+), and C1q (±) in the mesangium and along capillary loops. Due to institutional limitations at the time of biopsy, electron microscopy was not performed.

**Figure 4:**
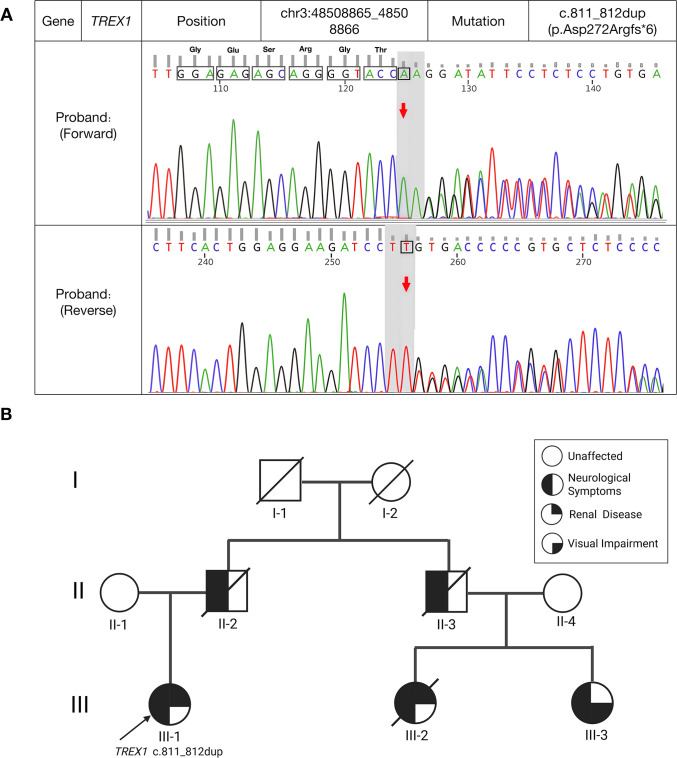
**(A)** Sanger sequencing identification of the *TREX1* frameshift mutation. Although the variant was initially detected by Whole-Exome Sequencing (WES), this chromatogram shows the Sanger sequencing confirmation of the heterozygous c.811_812dup mutation. The mutation results in a frameshift and a predicted truncated protein (p.Asp272Argfs*6). **(B)** Pedigree of the patient’s family. Squares indicate males; circles indicate females; diagonal lines indicate deceased individuals. The arrow indicates the proband (III-1). The genotype of the proband (*TREX1* c.811_812dup) is indicated below the symbol. Notably, genetic testing was only performed for the proband. Symbols are shaded to represent specific clinical phenotypes as follows: black left half indicates neurological symptoms; black upper-right quadrant indicates renal disease; black lower-right quadrant indicates visual impairment. Individuals with full shading exhibited multiple systemic manifestations. The clustering of these phenotypes across generations is consistent with an autosomal dominant inheritance pattern.

Genetic Testing: Given the distinctive neuroimaging, multisystem involvement (renal, brain, eye, peripheral nerve), and suspected TMA pathology, whole-exome sequencing (WES) was performed. It identified a heterozygous frameshift mutation in the *TREX1* gene (c.811_812dup, p.Asp272Argfs*6), which was confirmed by Sanger sequencing (**Figure 4A**). The family pedigree is consistent with an autosomal dominant inheritance pattern, although definitive genetic co-segregation was limited by the availability of samples from other family members (**Figure 4B**).

Final Diagnosis: Based on the clinical presentation, neuroimaging characteristics, pathological review, and genetic findings, the patient was diagnosed with genetically confirmed *TREX1*-associated retinal vasculopathy with cerebral leukoencephalopathy and systemic manifestations (RVCL-S) with an incomplete phenotype, overlapping with Systemic Lupus Erythematosus/Lupus Nephritis (SLE/LN) (13, 14).

Multidisciplinary Management: Given the complexity of the disease and multisystem involvement, a comprehensive multidisciplinary care plan was implemented:

Immunosuppression Strategy: To balance the control of LN activity against the risk of RVCL-S-related vascular damage, a low-intensity maintenance regimen was established: methylprednisolone 4 mg/d, mycophenolate mofetil 0.5 g/d, and hydroxychloroquine 0.2 g/d.

Cognitive & Neuropsychiatric Care: For moderate cognitive impairment (MoCA 13), a neuropsychiatrist assessed her mental status. Extensive safety education and caregiving guidance were provided to her family to manage cognitive decline.

Rehabilitation: Physiotherapists designed a daily bedside rehabilitation program focused on balance training and fall prevention to address gait instability.

Symptomatic & Supportive Care: Cervical spondylosis and neuropathic pain were managed conservatively with analgesics and physical therapy. Adjunctive medications included calcitriol (bone health), clopidogrel 75 mg/d (antiplatelet), and valsartan 80 mg/d (renal protection).

Outcome and Follow-up At the most recent follow-up in August 2025 (12 months post-discharge), the patient remained alive. Her neurological symptoms (gait instability and weakness) had stabilized with no further progression observed. Visual acuity and renal function also remained stable.

Patient Perspective: For years, I lived with uncertainty, struggling with leg weakness and unsteadiness that no one could fully explain. It was frustrating when treatments failed to stop my walking from getting worse. When genetic testing finally identified the *TREX1* mutation, I felt a sense of relief to know the true cause, even though I learned it is a rare genetic disorder. Although my walking ability has not fully recovered, I am grateful that my condition has stabilized over the past year. I hope that sharing my story will help doctors recognize this disease earlier in others, so they can get the right diagnosis sooner than I did.

## Discussion

This study documents the first known case of a patient with a *TREX1* frameshift mutation (c.811_812dup, p.Asp272Argfs*6) exhibiting both RVCL-S and LN (13, 14). The diagnosis of LN was confirmed by distinct pathological features (ISN/RPS Class IV) and serological markers, while the diagnosis of genetically confirmed *TREX1*-associated RVCL-S with an incomplete phenotype was validated by characteristic cerebral leukoencephalopathy and the pathogenic mutation. Whole-exome sequencing identified no additional pathogenic variants, suggesting that this dual phenotype likely arose from the identified heterozygous *TREX1* variant, although further familial co-segregation would strengthen this correlation.

The identified variant, *TREX1* c.811_812dup (p.Asp272Argfs*6), is a frameshift mutation in the C-terminal region. We queried the gnomAD database and confirmed that this variant is absent in the general population (Evidence level: PM2). According to ACMG/AMP guidelines, it is classified as Pathogenic (Evidence: PM2, PVS1, PP4). Mechanistically, this mutation results in a frameshift at codon 272, leading to the loss of the entire C-terminal transmembrane domain (residues 273–314). Although functional studies were not performed on this specific variant, previous experimental work by Orebaugh et al. demonstrated that a *TREX1* mutant truncated at residue 286 (*TREX1* 1-286) failed to localize to the endoplasmic reticulum (ER) and was diffusely dispersed throughout the cytosol (15). Since the p.Asp272Argfs*6 variant in our patient causes an even more extensive truncation, it is predicted to share this severe defect in subcellular localization.

Based on existing literature, we propose that this aberrant localization drives a dual pathogenic process, reflecting the complex genotype-phenotype correlations of *TREX1* mutations (16, 17). Unlike N-terminal mutations that cause loss of enzymatic function and interferonopathies (e.g., AGS), C-terminal truncating mutations retain catalytic activity but lose ER anchoring (18–20). First, the mislocalized protein cannot effectively degrade immunogenic DNA at appropriate subcellular sites, leading to cytosolic DNA accumulation. This likely triggers the cGAS-STING pathway, inducing a type I interferon response and autoantibody production that underlies the development of SLE/LN (7, 21). Second, the failure of *TREX1* to interact correctly with the ER oligosaccharyltransferase (OST) complex contributes to the non-inflammatory hyaline microvasculopathy characteristic of RVCL-S (22). Our patient’s renal biopsy uniquely captured this convergence: immune complex deposits (LN) coexisted with ischemic glomerular shrinkage (RVCL-S vasculopathy), suggesting these mechanisms may have acted synergistically to accelerate renal decline. Notably, emerging evidence indicates that mislocalized, catalytically active *TREX1* may accumulate in the nucleus, potentially leading to DNA damage and genomic instability, which provides a plausible explanation for the observed association between RVCL-S and elevated cancer risk (23, 24).

Traditionally, *TREX1* mutations have been linked to specific, isolated disease phenotypes. Pioneering research by Richards et al. demonstrated that C-terminal truncating mutations, despite maintaining catalytic activity, lead to protein mislocalization. This mislocalization is viewed as the primary mechanism driving the specific microvasculopathy associated with RVCL-S (2). However, the phenotypic spectrum may exhibit overlap. While the presence of autoantibodies has been reported in some RVCL-S patients (25), Wang et al. recently reported a patient with RVCL-S carrying the *TREX1* c.294dupA mutation who also met the classification criteria for SLE, presenting with cognitive impairment, brain calcifications, and retinal vasculopathy (12). Notably, their patient showed improvement in neurological symptoms, such as communication and ambulation, following treatment with methylprednisolone, mycophenolate mofetil, and hydroxychloroquine. In contrast, our patient experienced progressive neurological deterioration despite receiving a similar immunosuppressive regimen (prednisone, mycophenolate mofetil, and hydroxychloroquine). To better contextualize the novelty of our findings, we compared the clinical and renal characteristics of our patient with previously reported representative RVCL-S cohorts and overlap cases (**Table 1**). While renal dysfunction is a known feature of RVCL-S (3), it is typically ischemic in nature. Unlike the case reported by Wang et al. (12), which exhibited SLE features but spared the kidneys, our patient presents the first documented instance of biopsy-proven immune-complex mediated glomerulonephritis (LN) superimposed on the genetic background of RVCL-S. This discrepancy in therapeutic response suggests significant heterogeneity in the clinical presentation and treatment sensitivity of the RVCL-S/SLE overlap phenotype, indicating that immunosuppression alone may not be sufficient to arrest neurovascular progression in all patients.

**Table 1:** Comparison of clinical features, genetic findings, and renal characteristics between the current case and previously reported RVCL-S cohorts.

Study (reference)	*TREX1* variant	Clinical phenotype	Autoimmune features	Renal characteristics
Richards et al. (2007) (2)	V235fs (and others)	Classic RVCL-S	Generally negative or not detailed	Late-stage renal failure (Vasculopathy)
Stam et al. (2016) (3)	Various C-terminal frameshifts (n=78)	Classic RVCL-S	Autoantibodies reported in a subset, but full SLE rare	Renal dysfunction (~50-60%) (Ischemic/Vasculopathic)
Wang et al. (2024) (12)	c.294dupA (C99fs)	RVCL-S + SLE	Positive (ANA, anti-cardiolipin); met SLE criteria	Normal renal function; no evidence of nephritis.
Current Case	c.811_812dup (p.Asp272Argfs*6)	RVCL-S + SLE + LN	Positive (ANA, anti-dsDNA, low C3/C4)	Lupus Nephritis (Class IV) (Immune-complex mediated)

RVCL-S, Retinal Vasculopathy with Cerebral Leukoencephalopathy and Systemic Manifestations; SLE, Systemic Lupus Erythematosus; LN, Lupus Nephritis; ANA, Antinuclear Antibody; anti-dsDNA, anti-double stranded DNA; C3/C4, Complement components 3 and 4.

This case underscores a significant therapeutic challenge. The first-line immunosuppressants used for treating SLE/LN, especially high-dose glucocorticoids and calcineurin inhibitors, carry inherent risks that theoretically could exacerbate vascular endothelial injury and promote thrombosis (26, 27). The observed effects are hypothesized to potentially contribute to the worsening of the underlying microvasculopathy and ischemic progression associated with RVCL-S. On the other hand, reducing the intensity of immunosuppressive treatment to safeguard vascular function could lead to uncontrolled lupus nephritis and irreversible end-stage renal disease. This “double-hit” pathophysiology is proposed as a theoretical framework to help elucidate the patient’s inadequate response to standard immunosuppressive therapies. Consequently, managing patients with the RVCL-S/SLE-LN overlap demands meticulous care, aiming to strike a delicate balance between effectively suppressing autoimmunity and minimizing iatrogenic vascular damage. Hydroxychloroquine is recommended as a foundational and ongoing treatment. In addition to its immunomodulatory effects, hydroxychloroquine has demonstrated benefits in improving lipid profiles, providing antiplatelet activity, and offering potential endothelial protection, which collectively may reduce cardiovascular risk and deliver dual advantages for these patients (28, 29). When stronger immunosuppression is required, it is prudent to conduct a thorough evaluation of the benefit-risk ratio. Although agents with lower vascular toxicity like mycophenolate mofetil (MMF) are preferred, their potential impact on endothelial repair pathways necessitates ongoing monitoring. Conversely, long-term use of high-dose glucocorticoids and calcineurin inhibitors should be approached with caution and employed only when absolutely necessary. The ultimate goal is to establish therapies that address the underlying causes of the disease. This may involve neutralizing the toxic effects of the mutant *TREX1* protein or specifically targeting its downstream pathways, potentially through the use of STING or JAK inhibitors (30, 31). Furthermore, anifrolumab, a monoclonal antibody blocking the type I interferon receptor, warrants consideration. Given that *TREX1* dysfunction can drive a type I interferonopathy characteristic of SLE, anifrolumab could theoretically ameliorate the systemic autoimmune manifestations in such overlap cases, although its specific efficacy on the non-inflammatory vasculopathy of RVCL-S remains to be established.

### Study limitations

This study has several limitations. First, the lack of samples from additional family members precluded co-segregation analysis to further support the genotype–phenotype correlation. Second, we did not measure serum type I interferon levels or interferon-stimulated gene expression, preventing direct assessment of immune activation. Third, the relatively short follow-up limited characterization of the long-term disease course. Future work should recruit more relatives for co-segregation studies, incorporate functional evaluation of type I interferon signaling to clarify immunopathological mechanisms, and extend follow-up to better define disease progression and outcomes. Fourth, a notable limitation is the absence of characteristic retinal vasculopathy at the time of examination, despite the clear presence of a pathogenic *TREX1* mutation and typical neuroimaging findings. While the patient presently manifests the ‘VCL-S’ components (Cerebral Leukoencephalopathy and Systemic manifestations), the lack of the ‘R’ (Retinal) component may indicate an early clinical stage or a partial phenotype. The incomplete ophthalmologic follow-up remains a challenge in definitively confirming the full clinical syndrome at this point, necessitating long-term longitudinal ophthalmologic confirmation to track the potential evolution of retinal lesions.

## Conclusion

In conclusion, this case provides clinical and genetic evidence that a single mutation in the *TREX1* gene, p.Asp272Argfs*6, can drive the coexistence of two distinct disease entities: RVCL-S and SLE/LN. This finding highlights the intricate nature of monogenic disorders and the variability within their clinical manifestations. It emphasizes the need for clinicians to consider *TREX1*-associated disorders in the differential diagnosis when encountering patients with early-onset or atypical SLE/LN who also exhibit neurological symptoms such as leukoencephalopathy or cognitive impairment, alongside neuropsychiatric lupus. Timely genetic testing is highly recommended for accurate etiological diagnosis. Future research should aim to clarify the specific molecular mechanisms by which *TREX1* C-terminal mutations activate the interferon pathway in different cellular contexts such as vascular endothelial cells versus immune cells. Additionally, efforts should be made to develop targeted therapies that can effectively address both vascular and autoimmune pathological processes simultaneously.

## Data Availability

The original contributions presented in the study are included in the article/**Supplementary Material**, further inquiries can be directed to the corresponding author.
