# Supplementary material for: First case of TREX1 mutation-driven retinal vasculopathy with cerebral leukoencephalopathy and systemic manifestations coexisting with lupus nephritis: a case report and mechanistic discussion
Source: Front Immunol. 2026 Mar 19;17:1758743. doi: 10.3389/fimmu.2026.1758743 (PMC13043996; doi:10.3389/fimmu.2026.1758743)
Supplement: Supplementary file 1 [file DataSheet1.pdf]

Supplementary Materials for:

First Case of TREX1 Mutation-Driven Retinal Vasculopathy with Cerebral Leukoencephalopathy and Systemic Manifestations (RVCL-S) Coexisting with Lupus Nephritis: A Case Report and Mechanistic Discussion

Authors: Wenjie Hao†, Shu Zhai†, Qianqian Zhu†, Xuan Zhou, Tingting Shen, Wei He, Yuying Sun, Wenming Yang*, Yulong Yang*

Corresponding Authors: Wenming Yang (yangwm8810@126.com)

Yulong Yang (1418057112@qq.com)

Table of Contents:

1.Supplementary Table S1: Detailed Laboratory Investigations

2.Supplementary Figure S1: Clinical Photograph of Pes Cavus

3.Supplementary Figure S2: Fundus Photography

4.Supplementary Figure S3: Additional Immunofluorescence Findings

Supplementary Table S1. Detailed Laboratory Investigations of the Patient.

Category	Parameter	Result	Reference Range
Renal Function	Serum Creatinine (μmol/L)	118.1↑	41 – 73
	Blood Urea Nitrogen (mmol/L)	9.41↑	2.6 – 7.5
	Cystatin C (mg/L)	1.75↑	0.51 – 1.09
Lipid Profile	Triglycerides (mmol/L)	0.62	< 1.70
	Total Cholesterol (mmol/L)	4.51	3.00 – 5.70
	LDL-Cholesterol (mmol/L)	2.38	1.89 – 4.21
Autoimmunity	Anti-dsDNA	Negative	Negative
	ANA (Immunofluorescence)	Negative	Negative
	Anti-Sm / Anti-RNP	Negative	Negative
	ANCA (MPO/PR3)	Negative	Negative
Hematology	WBC ($\times 10^9$/L)	6.87	3.5 – 9.5
	Hemoglobin (g/L)	86↓	115 – 150
	PLT ($\times 10^9$/L)	227	125 – 350

Footnotes:

Abbreviations: ANCA, Antineutrophil Cytoplasmic Antibody; LDL, Low-Density Lipoprotein.

Note: Arrows (↑/↓) indicate values outside the reference range. Despite elevated renal function parameters (Creatinine, BUN, Cystatin C), the negative autoimmune panel and stable clinical presentation indicated a quiescent phase of Lupus Nephritis with chronic renal insufficiency, rather than an acute flare.

Supplementary Figure S1

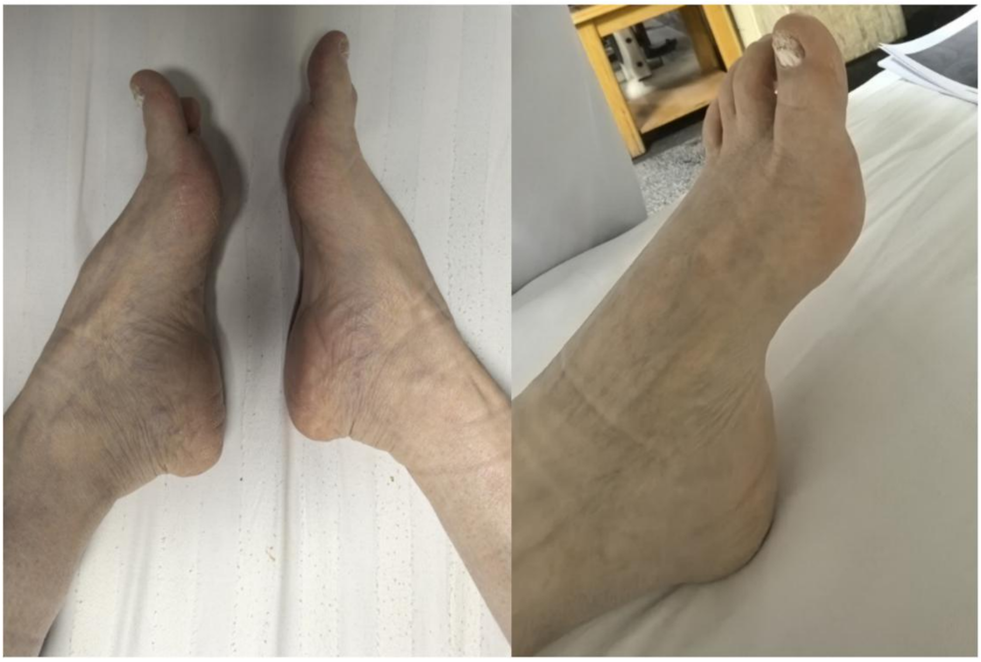

Figure S1. Clinical manifestation of peripheral neuropathy. The photograph demonstrates bilateral pes cavus (high-arched feet), a structural deformity often associated with long-standing hereditary motor and sensory neuropathies.

Supplementary Figure S2

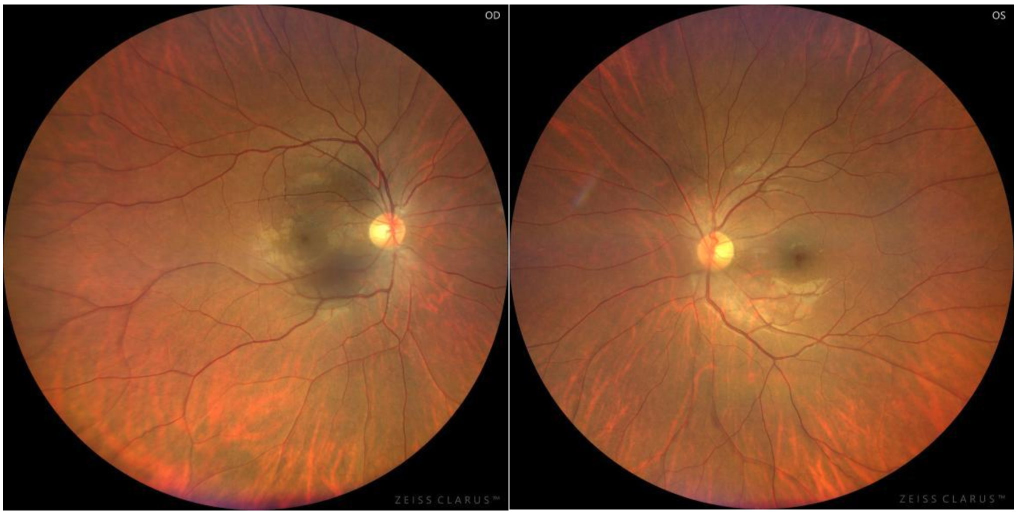

Figure S2. Fundus examination. Dilated fundus photography showing a normal optic disc and retinal vasculature, with no evidence of vasculitis, hemorrhage, or cotton-wool spots at the time of examination.

Supplementary Figure S3

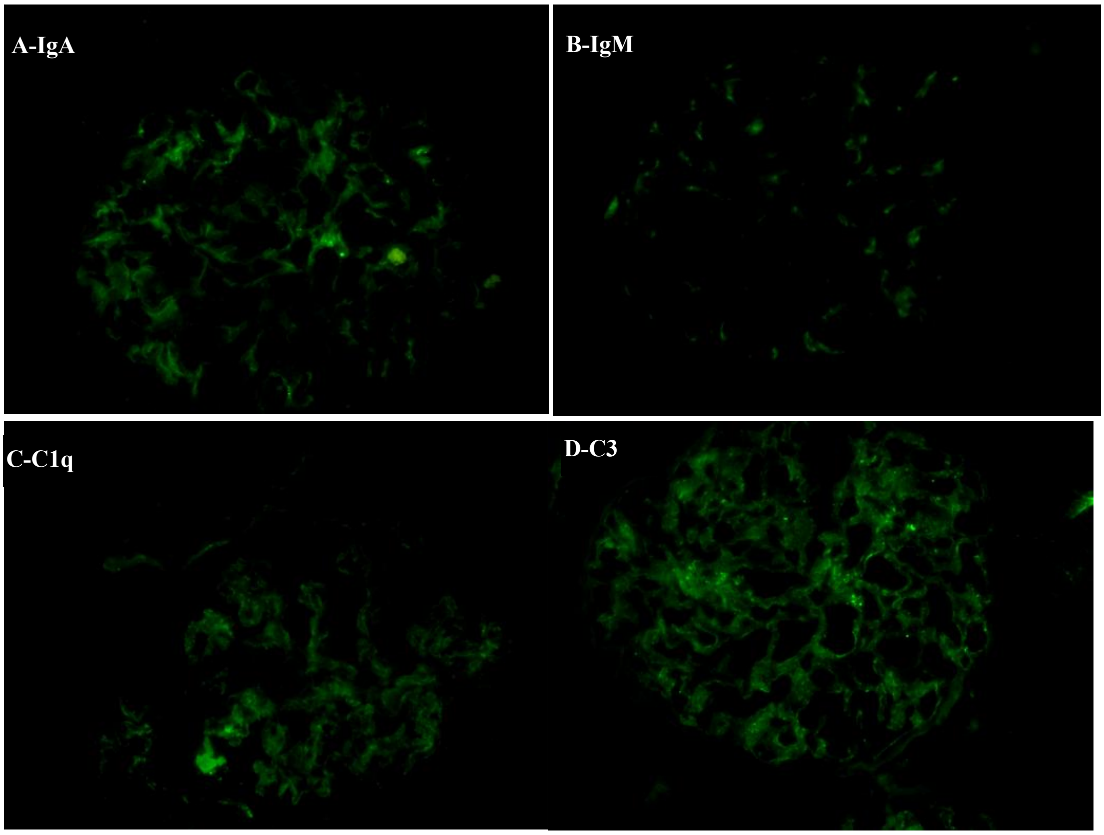

Figure S3. Additional Immunofluorescence Findings. Immunofluorescence microscopy demonstrates granular mesangial and capillary loop deposits of **(A)** IgA (+), **(B)** IgM (±), **(C)** C1q (±) ,**(D)** C3 (+).
